# EV-miRome-wide profiling uncovers miR-320c for detecting metastatic colorectal cancer and monitoring the therapeutic response

**DOI:** 10.1007/s13402-022-00688-3

**Published:** 2022-07-18

**Authors:** Chan-Keng Yang, Hung-Chih Hsu, Yu-Hao Liu, Wen-Sy Tsai, Chung-Pei Ma, Yi-Tung Chen, Bertrand Chin-Ming Tan, Ying-Yu Lai, Ian Yi-Feng Chang, Chi Yang, Chia-Yu Yang, Jau-Song Yu, Hsuan Liu

**Affiliations:** 1 Division of Hematology-Oncology, Department of Internal Medicine, Chang Gung Memorial Hospital at Linkou, Taoyuan, Taiwan; 2 Graduate Institute of Clinical Medical Sciences, College of Medicine, Chang Gung University, Taoyuan, Taiwan; 3 College of Medicine, Chang Gung University, Taoyuan, Taiwan; 4 Department of Cell and Molecular Biology, College of Medicine, Chang Gung University, Taoyuan, Taiwan; 5 Graduate Institute of Biomedical Sciences, College of Medicine, Chang Gung University, Taoyuan, Taiwan; 6 Division of Colon and Rectal Surgery, Chang Gung Memorial Hospital at Linkou, Taoyuan, Taiwan; 7 Department of Biomedical Sciences, College of Medicine, Chang Gung University, Taoyuan, Taiwan; 8 Molecular Medicine Research Center, Chang Gung University, Taoyuan, Taiwan; 9 Research Center for Emerging Viral Infections, Chang Gung University, Taoyuan, Taiwan; 10 Department of Neurosurgery, Lin-Kou Medical Center, Chang Gung Memorial Hospital, Taoyuan, Taiwan; 11 Department of Microbiology and Immunology, College of Medicine, Chang Gung University, Taoyuan, Taiwan; 12 Department of Otolaryngology-Head and Neck Surgery, Chang Gung Memorial Hospital at Linkou, Taoyuan, Taiwan; 13 Liver Research Center, Chang Gung Memorial Hospital at Linkou, Taoyuan, Taiwan

**Keywords:** Metastasis colorectal cancer, Small RNA sequencing, Small extracellular vesicles, miRome, Mesenchymal–epithelial transition

## Abstract

**Purpose:**

Molecular composition of circulating small extracellular vesicles (EVs) does not merely reflect the cells of origin, but also is enriched in specific biomolecules directly associated with the cellular transformation. However, while most of the currently identified EV-miRs are only geared towards one-dimensional disease detection, their application for long-term tracking and treatment response monitoring has been largely elusive.

**Methods:**

We established and optimized a rapid, sensitive and robust liquid biopsy sampling method, and further used small RNA sequencing to comprehensively catalogue EV-miRomes in association with the progression and outcome of metastatic colorectal cancer (mCRC).

**Results:**

By cross-comparison of EV-miRomes (n = 290) from multi-stage and longitudinal cohorts, we uncovered a 15-EV-miR signature with dual detection and long-term monitoring of tumor size progression for mCRC. From this panel, EV-miR-320c was uncovered as a strong clinical marker – aside from its diagnostic power and a therapeutic monitoring performance superior to carcinoembryonic antigen (CEA), its high expression has also been linked to lower overall survival and a greater likelihood of disease recurrence. Further, integrative analyses of tissue transcriptomic and liquid biopsy implicated this 15-EV-miR signature in programming the mesenchymal–epithelial transition (MET) for distant localization of the metastasized cells and also in creating a tumor-favoring metastatic niche.

**Conclusion:**

Our clinically-oriented delineation of the mCRC-associated circulating EV-miRomes systematically revealed the functional significance of these liquid biopsy markers and further strengthen their translational potential in mCRC therapeutic monitoring.

**Graphical abstract:**

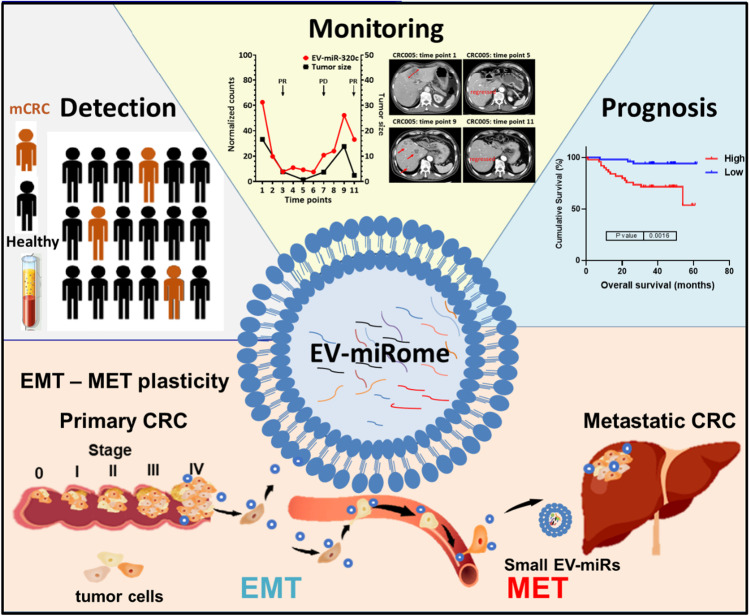

**Supplementary Information:**

The online version contains supplementary material available at 10.1007/s13402-022-00688-3.

## Introduction

mCRC is one of the leading causes of malignancy-related deaths worldwide, with a median overall survival rate at 30 months and a poor 5-year survival prognosis (only 10% of patients) [1, 2]. The current clinical strategy for mCRC primarily entails a combined treatment regimen of chemotherapy and targeted therapy, coupled with regular follow-ups for therapeutic response and disease outcome. While an increasing number of active agents have contributed to improved outcomes in patients with metastatic diseases, therapy resistance is emerging as a significant reason for limited therapeutic success. Therefore, it is a considerable clinical challenge to provide a timely and precise evaluation of the cancer curative effect, as well as monitoring of cancer recurrence/metastasis and patient survival. Currently, CEA and cancer antigen 19–9 (CA199) are the most widely used blood-based mCRC molecular markers. These biomarkers are valuable tools for disease status monitoring. However, the sensitivity of CEA and CA199 for CRC is low (47% and 14%, respectively), and the specificity is limited (80% and 89%, respectively), severely undermining their clinical use [3]. While contrast computed tomography (CT) of the abdomen is the gold-standard examination for tumor size and therapeutic response, it is laborious and costly. Owing to these existing challenges, a viable liquid biopsy platform that relies on small amounts of blood samples but provides a rapid and accurate assessment of mCRC progression, therapeutic response, and outcome is urgently needed.

Given that small EVs are functionally relevant entities in circulation, they have emerged in recent years as a promising target of liquid biopsies. Small EVs are membranous vesicles originating from the early endosomes-to-multivesicular bodies secretory pathway. Oncogenic stresses (such as epithelial–mesenchymal transition (EMT) and hypoxia) in tumor cells are known to promote small EV biogenesis via direct phosphorylation of specific components of the syntenin pathway [4, 5]. Upon formation, small EVs selectively enclose cellular molecules, such as lipids, RNAs, and proteins, and act as transporters that deliver these materials to recipient cells with tumorigenic implications. Consequently, small EV composition does not merely reflect the cells of origin, but is enriched in specific biomolecules directly associated with cellular transformation. In this capacity, small EVs may presumably be exploited as an effective and noninvasive means for detecting the presence of human cancers and even tracing their dynamic evolution. This notion is strongly supported by a recent global profiling of tumor-associated small EV proteins, which uncovered powerful biomarkers for cancer detection and unequivocal classification of primary tumor types [6]. By virtue of the tissue-specific and/or physiological state-specific patterns also exhibited by RNA expression, it could be equally advantageous to utilize small EV transcriptome signatures as a readout for the progression, metastasis, and outcome of malignancies. To this end, the establishment of a rapid, sensitive, and robust method for isolating and quantifying enclosed RNA molecules constitutes a critical prerequisite to small EV research and is therefore a key effort of our present study.

Several studies have shown that detection of specific EV-miRs may represent a novel diagnostic tool [3, 7–9]. Due to the high variability in the methods of sample collection and EV isolation, there have been large disparities in experimental outcomes among different studies. Of note, since these candidates are mainly used for detecting CRC, the application for long-term treatment monitoring has been largely absent. In this study, with the goal of cataloging a comprehensive small EV-miRome underlying mCRC, we implemented an improved and more robust liquid biopsy sampling method and optimized the experimental process of sample preparation for deep sequencing profiling. We analyzed small RNA profiles in EVs (n = 290) and uncovered a signature of 15 EV-miRs that can monitor both the metastatic progression of CRC and its long-term treatment outcome (15 mCRC-EV-miRs). Based on this panel, we further established EV-miR-320c as a strong clinical marker owing to its diagnostic power and association with overall survival and disease recurrence. Further, integrative analyses of tissue transcriptomic and liquid biopsy data provided strong evidence for the involvement of these 15 mCRC-EV-miRs in programming the MET signaling and consequently the distant localization of the metastasized cells.

## Materials and methods

### Collection of clinical specimens from patients with CRC

Clinical samples were collected from patients prospectively enrolled at the Chang Gung Memorial Hospital, Linkou, Taiwan, from December of 2014 to June of 2018. Patient follow-ups were regularly updated and clinical characteristics and statistics are summarized in Tables 1, 2, 4, and 5. Plasma samples were prepared by collecting 2 mL of fresh blood in blood collection tubes containing EDTA (with < 4 h storage at 4 °C) and centrifuging for 10 min at 1900 × g at 4 °C. The plasma (yellow) phase was transferred to a new tube, followed by additional centrifugation at 3000 × g, and stored in aliquots at − 80 °C. For the tissue sample preparation, a previously published procedure was implemented [10]. Each subject or the closest relative provided written informed consent for the study, which had been approved by the institutional review board (IRB) and ethics committee of Chang Gung Memorial Hospital (IRB 201601135B0 and IRB 103-2529B).

**Table 1:** Demographics of mCRC discovery cohort

	Discovery cohort (n = 48)		
	mCRC (n = 18)	healthy (n = 30)	*p* value
Age in years, mean (SD)	63.2 (12.3)	62.8 (12.1)	0.928^a^
Range	41–80	38–74	
Gender, (%)			> 0.99^b^
Male	12 (66.7)	20 (66.7)	
Female	6 (33.3)	10 (33.3)	

^a^two sample t-test

^b^Fisher's exact test

**Table 2:** Sampling time points for mCRC specimens

Patient	treatment-naïve	post-treatment sampling											
	1	2	3	4	5	6	7	8	9	10	11	12	13
CRC001	CRC00101	CRC00102	CRC00103	CRC00104	CRC00105	CRC00106	CRC00107	CRC00108					
CRC002	CRC00201	CRC00202	CRC00203										
CRC003	CRC00301	CRC00302	CRC00303	CRC00304	CRC00305	CRC00306		CRC00308	CRC00309	CRC00310	CRC00311	CRC00312	CRC00313
CRC005	CRC00501	CRC00502	CRC00503	CRC00504	CRC00505	CRC00506	CRC00507	CRC00508	CRC00509		CRC00511		
CRC006	CRC00601	CRC00602	CRC00603	CRC00604	CRC00605	CRC00606							
CRC007	CRC00701	CRC00702		CRC00704	CRC00705								
CRC008	CRC00801												
CRC009	CRC00901												
CRC010	CRC01001	CRC01002	CRC01003										
CRC011	CRC01101	CRC01102		CRC01104	CRC01105	CRC01106	CRC01107	CRC01108					
CRC012	CRC01201		CRC01203										
CRC013	CRC01301	CRC01302	CRC01303	CRC01304									
CRC014	CRC01401	CRC01402	CRC01403										
CRC015	CRC01501	CRC01502	CRC01503	CRC01504	CRC01505	CRC01506	CRC01507	CRC01508	CRC01509	CRC01510	CRC01511	CRC01512	CRC01513
CRC016	CRC01601	CRC01602	CRC01603	CRC01604	CRC01605	CRC01606							
CRC018	CRC01801	CRC01802	CRC01803		CRC01805			CRC01808	CRC01809				
CRC026	CRC02601	CRC02602				CRC02606							
CRC031	CRC03101	CRC03102	CRC03103	CRC03104	CRC03105	CRC03106							

### Purification, characterization, and analyses of circulating small EVs derived from metastatic CRC patients’ plasma

Circulating small EVs were isolated from 1 mL of pre-filtered plasma using a procedure described in the exoEasy Plasma Handbook (QIAGEN) [11]. Briefly, plasma was mixed with binding buffer (XBP) and added to the exoEasy membrane affinity column for binding. After centrifugation, the flow-through was discarded and wash buffer (XWP) was added to the column to wash off nonspecifically retained materials. Subsequently, the spin column membrane was incubated with elution buffer (XE) for 5 min and centrifuged for 5 min at 500 × g to collect the eluted circulating small EVs. Circulating small EV preparations were verified by electron microscopy (JEM-2100 Plus) and further analyzed for vesicle size and particle number using the NS300 nanoparticle characterization system (NanoSight, Malvern Instruments) equipped with a blue laser (488 nm). For protein analysis, circulating small EVs were concentrated using ultracentrifugation at 120,000 × g for 4 h and subjected to Western blot analysis of known vesicle-enriched proteins, including CD9 (Invitrogen, 10626D), Syntenin-1 (Proteintech, 22,399–1-AP), and a negative marker of cellular contamination, Calnexin (Proteintech, 10,427–2-AP).

### Cell Culture

Both HT-29 and HCT116 colorectal carcinoma cell lines were cultured in McCoy’s 5A medium supplemented with 10% heat-inactivated fetal bovine serum and 1 U/mL penicillin–streptomycin. All culturing reagents were purchased from Thermo Fisher Scientific. Cells were maintained at 37 °C with 5% CO_2_ in a humidified incubator. One day before EV isolation from the medium, the cells were shifted to serum-free condition for 24 h. Six milliliters of the medium were harvested, subjected to centrifugation to remove cell debris, and subsequently purified for EV RNAs as outlined below.

### RNA purification from circulating small EV

After EVs were bound on the exoEasy membrane affinity column as described in the isolation procedure above, the vesicles were lysed by adding QIAzol to the spin column and further collected by centrifugation (exoRNeasy, QIAGEN). Following chloroform extraction, samples were thoroughly mixed and centrifuged to separate organic and aqueous phases. The aqueous phase was recovered and mixed with ethanol. The sample-ethanol mixture was added to a RNeasy MinElute spin column and centrifuged. The column was washed once with buffer RWT and then twice with buffer RPE followed by elution of RNA in water. The RNA concentration, purity, and integrity were assessed using the RNA Nano 6000 Assay Kit of the Agilent Bioanalyzer 2100 System (Agilent Technologies, CA, USA).

### Small RNA library construction and sequencing

Prior to sequencing experiments, RNA concentration, purity, and integrity were assessed using the RNA Nano 6000 Assay Kit of the Agilent Bioanalyzer 2100 System (Agilent Technologies, CA, USA). Illumina sequencing libraries were prepared using the NEXTflex™ small RNA-seq kit v3 Guide (Bioo Scientific, 5132–05), according to the manufacturer’s instructions. For each library, 60 ng of purified small EV RNA were ligated to 3’ and 5’ adaptors and then reverse transcribed to cDNA using adaptor-specific primers. After purification and PCR amplification using universal and specific barcode primers, the miRNA library was resolved and recovered from the 6% TBE-PAGE gel based on the corresponding size for miRNA distribution. The yield and size distribution of the small RNA libraries were assessed using the Agilent 2100 Bioanalyzer instrument with the High-Sensitivity DNA Assay (Agilent Technologies). Equal concentration of each library was sequenced on a NextSeq 500 (Illumina) platform.

### Sequencing data analysis and bioinformatics analysis

Upon completion, sequencing data were assessed for quality and trimmed of the primer-adaptor sequences by the Partek® Flow® Genomic Analysis Software (Partek), followed by alignment to the human reference genome (hg38) (by bowtie). After annotation of known miRNA based on miRBase, the number of reads for each miRNA was normalized to reads per million (RPM) and quantified across all samples. The statistical package of Partek® Flow® Genomic Analysis Software was used to yield differential expression, volcano plot, and hierarchical clustering analyses. miRNA target prediction was compiled by the microRNA Target Filter analysis module of Ingenuity Pathway Analysis (IPA, QIAGEN), while target pathway annotations were done by the Core Analysis of IPA.

### RNA extraction and quantitative reverse transcription PCR (RT-qPCR)

Total RNA from cells was isolated by the TRIzol reagent (Thermo Fisher Scientific) according to manufacturer’s instructions. For miRNA RT reaction, 1 μg of total cellular RNA or 150 ng of EV RNA was first converted into cDNA by Superscript III reverse transcriptase (Invitrogen), in which miRNA-specific stem-looped RT primers were used (miR-320c: CTCAACTGGTGTCGTGGAGTCGGCAATTCAGTTGAGACCCTCTCAAC; U6: CTCAACTGGTGTCGTGGAGTCGGCAATTCAGTTGAGAAAATATGGAACG). For the quantitative determination of the reversely transcribed products, real-time PCR was performed on an Applied Biosystems 7500 Fast PCR System (Foster City, CA, USA) using the standard SYBR green method. For expression analysis, the Ct values of miRNA were first normalized to U6 genes in the same samples. The resulting ∆Ct was further utilized to assess the relative gene expression between different experimental groups, with the data presented as fold change to control. Sequences of primers used in real-time PCR assay are as follows: miR-320c, CGGCGGAAAAGCTGGGTTGAGAG; U6, CAAATTCGTGAAGCGTTCCA; universal reverse, CAACTGGTGTCGTGGAGTCGG.

### Statistical analysis

The two-sample t-test and Fisher’s exact test were employed for the analysis of clinical factors within the cohort. The Kaplan–Meier method was used to determine marker association with overall and progression-free survival. Diagnostic accuracy of candidate EV-miRs was assessed by receiver operating characteristic (ROC) curves analysis, and the area under the ROC curve (AUC) was calculated. Pearson’s correlation coefficient analysis was used to evaluate the correlation among EV-miRs expression, tumor size, and CEA and CA199 levels. Statistical significance of the shown comparisons was set at p < 0.05. All analyses were performed using the Statistical Package for the Partek® Genomics Suite® statistical software and SPSS (SPSS-Science, Chicago, IL, USA).

## Results

### Establishment of standardized procedure for small EV-miRome characterization

Previous reports have raised the notion that different preparation methods of plasma samples could result in variable compositions of circulating small EVs and thus inconsistent profiles [11]. Prior to small EV isolation, the most commonly practiced plasma preparation in a hospital setting is done by low-speed centrifugation and subsequent storage of plasma at − 80 °C. This method is problematic regarding profiling cell-free fluids. The potential issues include (1) insufficiently removed cells and debris that compromise sample quality upon storage and (2) prolonged storage of partially purified plasma might result in additional background generated in vitro from blood cell-derived vesicles. To achieve robust sampling in the clinical application in a consistent manner, we developed a refined approach by incorporating the following modifications: First, the centrifugation-based removal of cellular materials was performed immediately after blood collection (< 4 h, at 4 °C), which aimed to lower the risk of additional background from blood cell-derived vesicles generated in vitro. Second, to fully exclude cell contamination, we included an initial low g-force centrifugation step to separate cells from plasma, followed by an additional higher g-force centrifugation step to remove all remaining cellular debris. This two-step centrifugation is critical because, given the much higher abundance of RNA in the cells (by several orders of magnitude), even small amounts of cellular debris could have a significant effect on RNA profiling of cell-free fluids. Third, to isolate small EVs from stored plasma, we employed the previously reported membrane-based affinity binding step, which provides rapid isolation of small EV RNAs within 1 h [11]. This adjustment was intended to address the low recovery and time-consuming steps imposed by the conventional centrifugation-based approach while maintaining high purity in isolation.

**Fig. 1:**
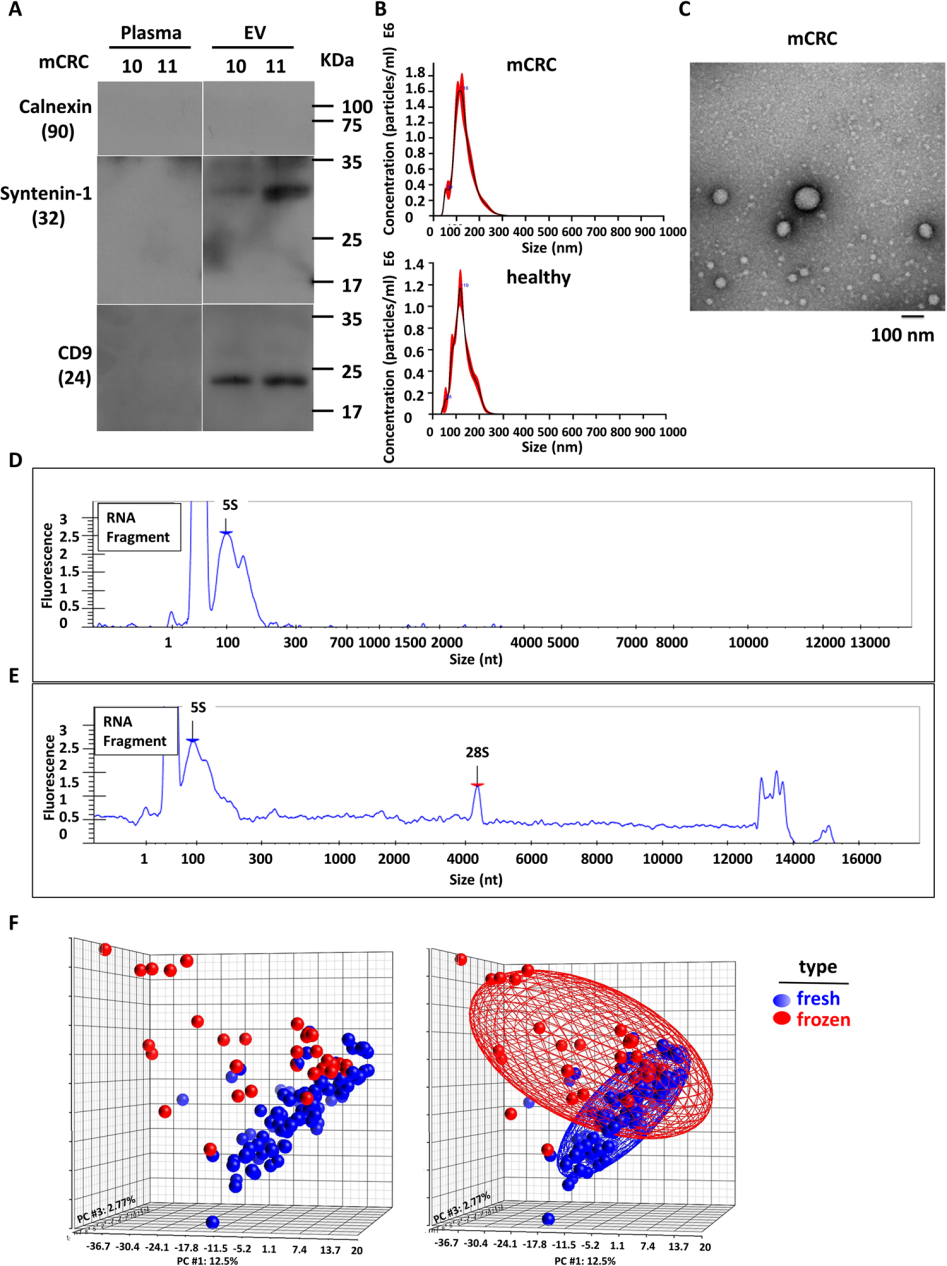
Establishment and characterization of standardized procedure for circulating small EV isolation. Small RNA sequencing was used to compare different plasma preparation methods of purifying circulating EV-miRs. RNAs were isolated by using two types of plasma small EVs preparation methods. (**A**) EV and corresponding plasma samples were prepared from two mCRC patient specimens (patients 10 and 11). Western blot was performed to analyze the small EV protein markers including CD9 and Syntenin-1, and a negative marker of cellular contamination, Calnexin. (**B**) NanoSight data of microvesicles eluted from the membrane affinity column. (**C**) Scanning electron microscopy analysis of CRC patients’ circulating small EVs. (**D-E**) RNA size distribution for fresh (**D**) and hemolytic (**E**) blood samples. (**F**) Small RNA-sequencing experiments were performed. Upon read alignment, miRNA expression levels were determined based on the normalized read count values. PCA plots are shown to depict the distributions of miRNA expression profiles in fresh (blue) vs. frozen (red) plasma samples. Significant separation of the two groups is indicative of distinct transcriptome signatures

Small EVs isolated from mCRC patient plasmas by membrane-based affinity was confirmed by Western blot analysis of known vesicle-enriched markers CD9 and Syntenin-1 and a negative marker of cellular contamination, Calnexin (Fig. 1A). By using the NanoSight instrument and scanning electron microscopy (Fig. 1B and C), vesicle structures in the expected size range of 50 ~ 200 nm were clearly visible. Next, after RNA extraction from the small EVs, RNA quality was monitored by the Caliper instrument. Figure 1D shows that the RNA size distribution was mostly less than 200 nt. In low-quality or hemolytic samples, the same quality control experiment would yield a relatively higher abundance of 18S and 28S (Fig. 1E), indicating contamination by intracellular RNA. This type of sample will be excluded from our subsequent analyses and studies.

To evaluate the performance of our optimized method entailing two-step centrifugation in fresh plasma sample preparation, we compared its small RNA profiles to that of the traditional version (i.e., initial centrifugation followed by storage). Small RNA-sequencing experiments consequently revealed that miRNA transcriptomes are considerably different between the two types of preparations (Fig. 1F): the PCA plots clearly illustrated that inter-sample variances were much greater in the traditional method than in our optimized method (red vs. blue). Viewed together, our results shown here highlighted the importance of a standardized protocol for sample processing to minimize the effect of sampling workflow on the eventual data outcome.

### Patients and samples

We collected 290 blood specimens from CRC patients and healthy controls at the Chang Gung Memorial Hospital in Taiwan, and prepared the samples following the standard method described above. For the first discovery cohort, Table 1 shows the basic clinical information of 48 samples including mCRC and healthy controls. Sixteen of the mCRC patients were recorded longitudinally with available post-treatment blood samples every 6 weeks during chemotherapy and/or targeted therapy (Table 2). We then completed the small RNA sequencing of all the available liquid specimens from this patient group. Consequently, there were 98 sequencing datasets, from which we generated an EV-miRome database of mCRC patients. We also recorded in detail patient response in terms of CEA and CA199 readings, tumor size, and potential treatment resistance after long-term treatment (Table S1). To complement the discovery cohort, we also collected 102 CRC patients of different stages, together with 90 healthy controls, totaling 192 samples in the validation cohort for further analysis and confirmation (Table 4). Moreover, for comparing the miRomes between circulating small EVs and solid tumor tissues, we incorporated a previously published miRNA sequencing dataset of 102 matched pairs of CRC tumor and adjacent normal specimens (Table 7) [10, 12]. In summary, this extensive specimen collection with 290 EV-miRome and 204 tissue miRomes constitute a strong and comprehensive molecular database, facilitating further interrogation of mCRC progression and therapeutic response.

### Identification of differentially expressed small EV RNA in pretreated mCRC patients

**Fig. 2:**
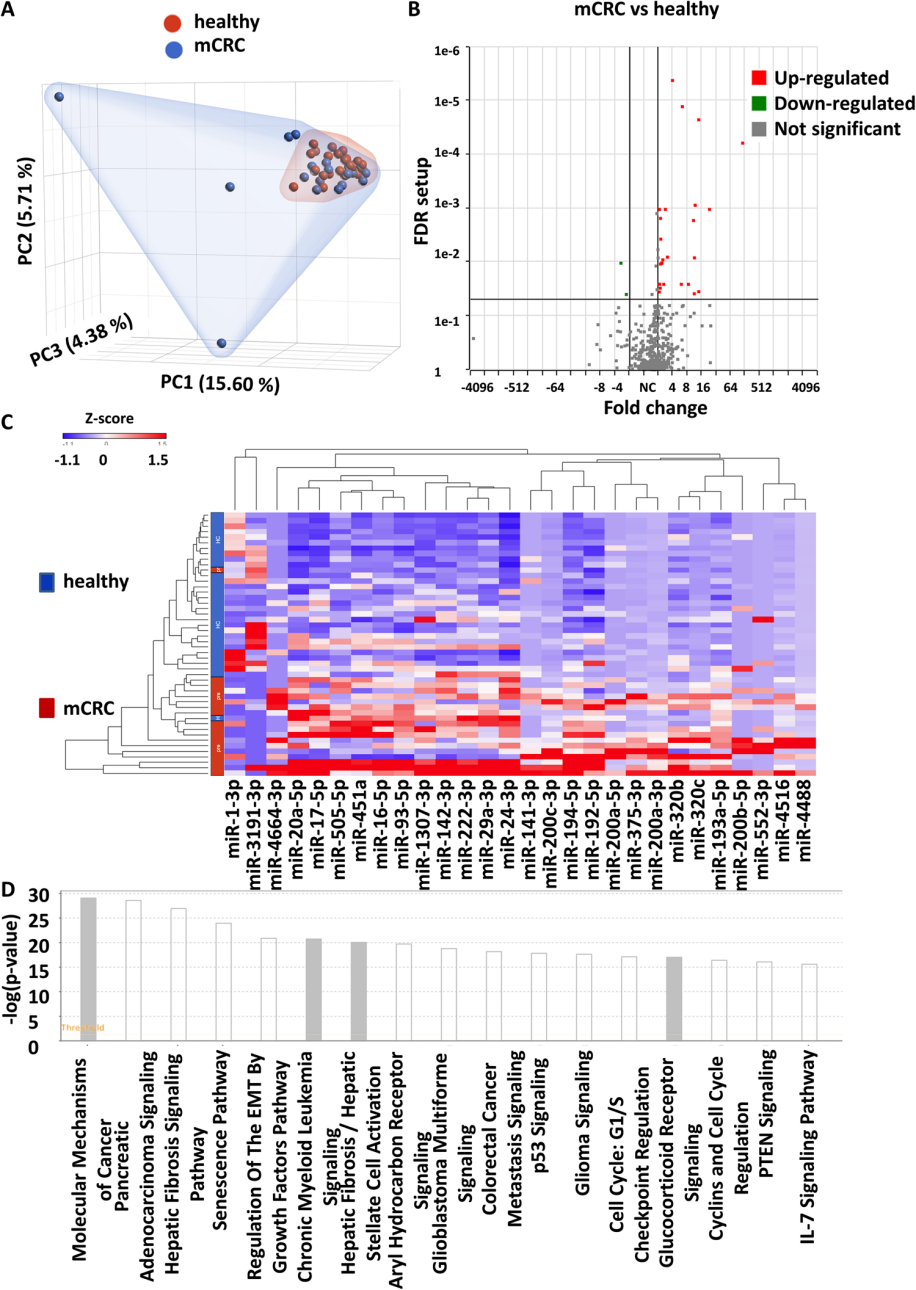
Systematic profiling for small EV RNAs differentially expressed in mCRC patients. Small RNA-seq was performed on 18 mCRC patients and 30 healthy controls. (**A**) PCA plot depicts the distributions of EV-miR expression in healthy (red) vs. mCRC (blue). (**B**) Volcano plot depicts the distribution of DE-EV-miRs. EV-miRs significantly upregulated (red) or downregulated (green) in the mCRC vs. healthy are marked accordingly. (**C**) Hierarchical clustering analysis of the selected 28 DE-EV-miRs in mCRC. (**D**) IPA pathway analysis of 586 target mRNA genes potentially under regulation by the EV-miRs. The colors of the bars correspond to the possibility of whether the activity of the enriched pathway could be predicted (white, yes; gray, no)

We first performed differential expression profiling of EV-miRs between pretreated mCRC patients and healthy subjects in the discovery cohort (Table 1). We identified 1,058 EV-miRs. PCA plot analysis was then used to depict the overall distribution of miRNA profiles, revealing a broader pattern of distribution for the mCRC patient group in comparison to normal subjects (Fig. 2A). This molecular distinction was in line with the typically heterogeneous characteristics of malignant tissues. By using the criteria of FDR < 0.05 and > 2 × fold changes in expression, we subsequently uncovered 28 differentially expressed EV-miRs (DE-EV-miRs) in the mCRC vs. healthy comparison, of which 26 miRNAs were upregulated and 2 were downregulated in the mCRC group (Fig. 2B and Table S2). The hierarchical clustering analysis based on the DE-EV-miRs showed distinct expression patterns between the two groups, with close correspondence to disease status (Fig. 2C). To evaluate the early diagnostic value of these candidate EV-miRs, we used the ROC curve analysis to reveal that 8 EV-miRs with ROC with more than 0.85 (EV-miR-200c-3p, EV-200a-3p, EV-miR-4488, EV-miR-194-5p, and EV-miR-4516, EV-miR-320c, EV-miR-193a-5p, and EV-miR-375-3p) (Table S2 and Fig. S1). Further, by using IPA Target Filter analysis, we found that these 28 EV-miRs could target 586 mRNA genes based on previously annotated experimental evidence. Further IPA canonical pathway analysis revealed an enrichment of genes with functional implications in the Molecular Mechanisms of Cancer, Regulation of the Epithelial–Mesenchymal Transition Pathway and Colorectal Cancer Metastasis Signaling (Fig. 2D).

### EV-miR-320c in circulating small EVs served as a biomarker for detecting mCRC and monitoring therapeutic response

**Fig. 3:**
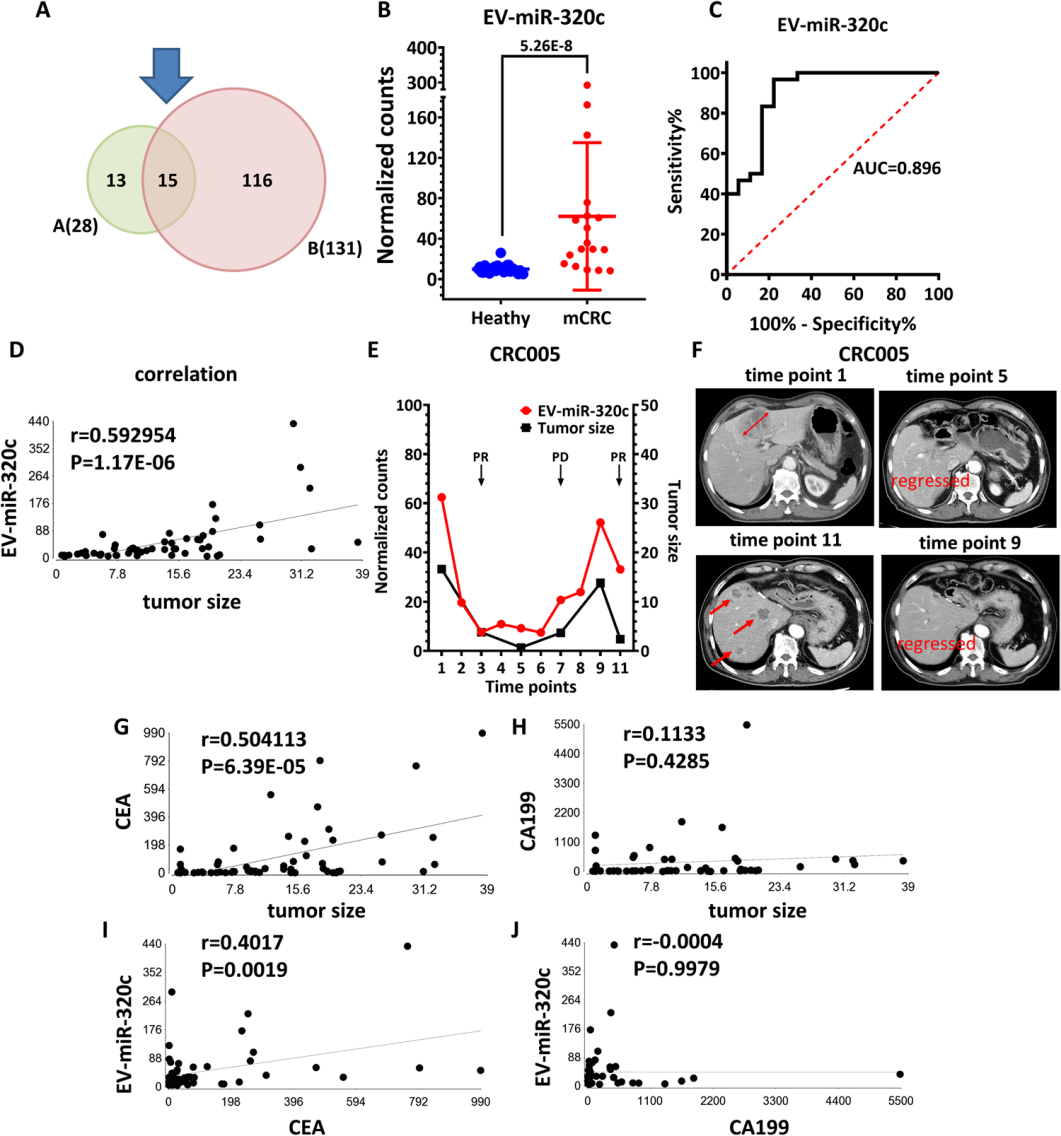
Identification of EV-miRs with dual potential of detecting both mCRC and long-term treatment outcome. (**A**) Venn diagram showing sizes and overlap of EV-miR datasets identified for mCRC detection (left) and for treatment response monitoring (right). (**B**) EV-miR-320c as an example of the mCRC-associated DE-EV-miRs in our mCRC vs. healthy comparison. (**C**) ROC curve analysis of EV-miR-320c as an indicator of mCRC revealed an AUC value of 0.896. (**D**) Pearson’s correlation test for the relationship between EV-miR-320c and tumor size. (**E**) The correlation of EV-miR-320c expression (left axis; red curve) with tumor size changes (right curve; black curve) in CRC patient 5 (CRC005). (**F**) CT images of patient 5 at the indicated time points during the treatment course, which show the progression of disease in response to treatment. Red arrows denote the locations of tumor detection. (**G-J**) Evaluation of EV-miR-320c as a monitoring marker for tumor size and comparison with CEA and CA199. The degree of correspondence between selected marker expression and tumor size was determined by Pearson’s correlation test. Pairwise comparison was done between (**G**) CEA and tumor size, (**H**) CA199 and tumor size, (**I**) CEA and EV-miR-320c, and (**J**) CA199 and EV-miR-320c

To identify effective EV-miRs with the purpose of real-time monitoring of treatment response, we rely on the serial sampling collected (Table 2) and the corresponding sequencing data. In the 98 EV-miRome datasets, there are 58 samples with clinical tumor size information (Table S1). By integrating clinical data, we carried out a correlation analysis (i.e., Pearson’s correlation test) to pinpoint candidate EV-miRs with expression profiles closely coordinated with tumor sizes. We then identified 131 EV-miRs with expression levels correlated with the tumor sizes (with p value < 0.05, Table S3). Next, we cross-referenced with the dataset revealed in the Fig. 2C, leading to the identification of 15 EV-miRs with dual functions of detecting both mCRC and long-term treatment outcome (15 mCRC-EV-miRs) (Fig. 3A and Table 3). Among these dual-functional 15 mCRC-EV-miRs, EV-miR-320c is a promising candidate, with significant upregulation (6.29 ×) in the mCRC with AUC value of 0.896 (Fig. 3B, C and Table 3). Moreover, EV-miR-320c shows a significantly positive correlation with tumor size with high efficiency (r = 0.59, p = 1.17E-06) (Fig. 3D). As examples of correlation profiles, the correlation of EV-miR-320c expression with the tumor size changes in CRC patient 5 (CRC005) is shown in Fig. 3E. To this end, both EV-miR-320c underwent expression reduction initially during the partial response period of the first-line treatment but conversely upregulated when a relapse in disease occurred. After switching to the second-line treatment, which was accompanied by a partial response, there was evident decrease in the expression of EV-miR-320c. An example of CT images demonstrating the initial partial response (in terms of tumor size) observed for one of the patients (patient CRC005) is shown in Fig. 3F. The displayed follow-up times started from the time of pretreatment sample (time point 1) and progressed throughout the follow-up (up to time point 11). The correlations of EV-miR-320c expression with the tumor size changes in the other 15 CRC patients are shown in the Fig. S2. Collectively, our data indicated that EV-miR can be a powerful indicator of multiple clinical attributes, such as mCRC detection and real-time monitoring of treatment response.

**Table 3:** 15 mCRC-EV-miRs associated with mCRC detection and therapeutic response

Gene ID	r (Pearson's correlation)	p-value (Pearson's correlation)	ROC (mCRC)	Fold change (pre vs. HC)	P-value (fold change)
EV-miR-320c	0.593	1.1735E-06	0.896	6.3	5.2644E-08
EV-miR-4516	0.579	2.3759E-06	0.939	10.8	3.7395E-05
EV-miR-320b	0.558	6.6156E-06	0.743	2.4	0.0004
EV-miR-552-3p	0.499	7.9085E-05	0.683	6.0	0.0014
EV-miR-4488	0.470	0.0002	0.946	112.8	4.9997E-07
EV-miR-200b-5p	0.422	0.001	0.717	8.4	0.0012
EV-miR-193a-5p	0.421	0.001	0.894	2.8	1.6426E-05
EV-miR-4664-3p	0.383	0.003	0.742	11.2	0.0025
EV-miR-141-3p	0.341	0.010	0.779	11.3	0.0003
EV-miR-1307-3p	0.323	0.014	0.831	2.3	9.0321E-05
EV-miR-194-5p	0.313	0.018	0.943	3.9	8.6146E-09
EV-miR-200c-3p	0.303	0.022	0.954	11.7	8.8483E-06
EV-miR-29a-3p	0.277	0.037	0.769	2.2	0.0017
EV-miR-222-3p	0.270	0.042	0.750	2.1	0.0022
EV-miR-24-3p	0.269	0.043	0.841	2.1	1.4288E-05

Finally, to evaluate the prognostic effectiveness and clinical value of these markers, we assessed the power of EV-miR signatures in detecting tumor size relative to standards CEA and CA199. Consequently, our results demonstrated that CEA displays a correlation coefficient of 0.5041 with tumor size (p = 6.39E-05), whereas no significant correlation was evident for CA199 with tumor size (Fig. 3G and H). For the 15 EV-miRs identified by our approach, 3 (EV-miR-320c, EV-miR-4516, and EV-miR-320b) were with better correlation with tumor size than CEA (Table 3). In particular, we also discovered that EV-miR-320c exhibits a moderate expression correlation with CEA but not with CA199 (Fig. 3I and J), which is the first reported case of CEA-coordinated EV-miR. In summary, our studies in this part established the potential use of EV-miRs as dual-role markers for mCRC detection and treatment response monitoring.

### Independent validation of EV-miR-320c as a novel, specific biomarker for tumor progression and patient outcome

**Fig. 4:**
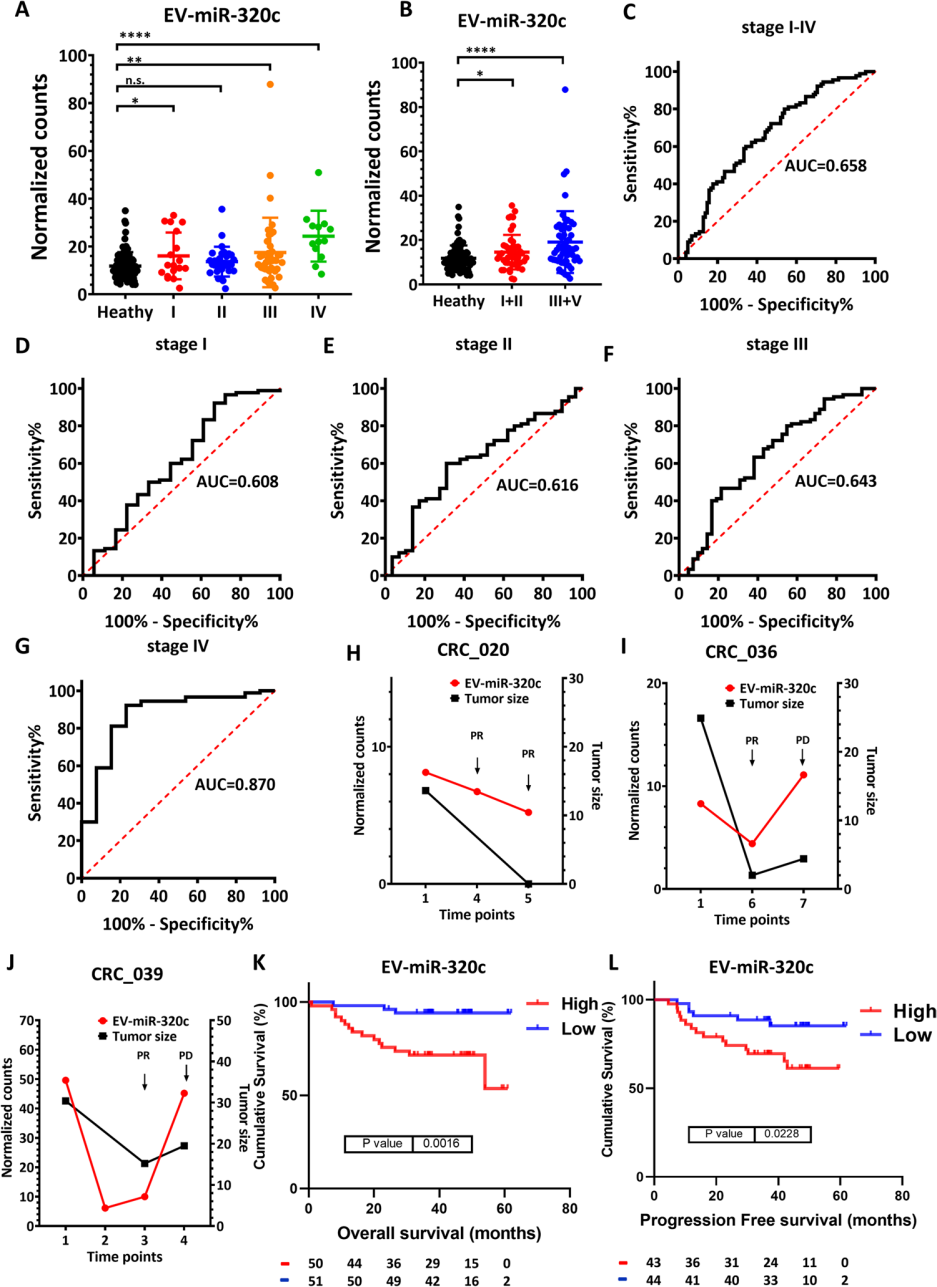
EV-miR-320c is a novel, specific biomarker for interpreting multiple clinical attributes of CRC. (**A**) The expression patterns of EV-miR-320c in healthy subjects and CRC patients of all four stages, as determined by small RNA-seq. (**B**) Alternative representation of the expression profiles of EV-miR-320c in healthy subjects and CRC patients of early stages (stage 1 + II) or late stages (stage III + IV). (**C-G**) ROC curve analysis for the power of EV-miR-320c expression in distinguishing all-stage CRC (**C**) and stage I-IV (**D-G**). (**H-J**) The expression correlation of EV-miR-320c with tumor size changes in CRC patients 020, 036, and 039. (**K & L**) Based on the expression levels of EV-miR-320c, overall survival analysis for stage I-IV CRC (**K**) and progression-free survival analysis for stage I-III CRC (**L**) were performed to illustrate the clinical association of EV-miR-320c to patient outcome

To independently verify the potential of EV-miR-320c in detecting and monitoring the therapeutic response in mCRC, we established additional EV-miRome datasets encompassing treatment-naïve CRC patients from all four stages. In this validation cohort, we collected 192 specimens from 102 CRC patients and 90 healthy controls (Table 4). We identified 1,248 EV-miRs. On average, 402 types of microRNAs are detected per sample (Fig. S3A, CRC, 407; healthy, 399), the highest expression is from EV-miR-451a, and the top 20 are shown in Table S4. In comparison with the healthy controls, the expression of the EV-miR-320c was significantly higher in the late stages of CRC (Fig. 4A, based on data from stages I to IV; Fig. 4B, based on grouping into low stages of I + II and advanced stages of III + IV). We next sought to determine the diagnostic value of EV-miR-320c in distinguishing all-stage CRC (Fig. 4C) and discovered that it was not quite significant (AUC = 0.658). In terms of distinguishing individual stages, we found that EV-miR-320c is specific only for mCRC (or stage IV CRC) with AUC of 0.870 (Fig. 4D to G), which is closely in line with the earlier mCRC cohort findings. We also enrolled three more patients with available post-treatment blood samples every 6 weeks during their therapeutic course. The change in the expression of EV-miR-320c across these samples was correlated with the therapeutic response (i.e., tumor size) as shown in Fig. 4H to J. Viewed together, these clinical profiles again demonstrated that EV-miR-320c has a dual function for detecting late-stage CRC and monitoring therapeutic response.

**Table 4:** Demographics of the validation cohort

Clinical stage (TNM)	Validation cohort (n = 192)				
	Healthy (n = 90)	CRC (n = 102)			
		I	II	III	IV
Patient numbers	90	18	29	42	13
Age in years, mean (SD)	57.24(10.37)	63.61(8.73)	60.79(10.31)	59.76(8.85)	62.31(9.52)
Range	35–74	46–77	37–79	37–76	46–77
Gender, n (%)					
Female	43(47.8)	4(22.2)	13(44.8)	19(45.2)	4(30.8)
Male	47(52.2)	14(77.8)	16(55.2)	23(54.8)	9(69.2)

To further strengthen the clinical relevance of the metastasis-associated EV-miRs, we then analyzed the association of the individual EV-miRs with patient survival and recurrence outcome. EV-miRome-wide analysis on EV-miR genes with detectable expression in > 50% of the cohort samples (384 EV-miRs) then uncovered 20 EV-miRs with significant connection to patient overall survival (Kaplan and Meier analysis, p < 0.05, Table 5). Of note, EV-miR320c was the most significant marker (p = 0.0016, Fig. 4K) – higher expression of EV-miR-320c was found to be associated with poor overall survival rate. In addition, among the 102 CRC EV-miRome datasets, there were 87 EV-miRome datasets of stage I-III CRC patients with disease recurrence records. To this end, further analysis pinpointed 14 EV-miRs associated with patient disease-free survival (Kaplan and Meier analysis, p < 0.05, Table 6). Expression of EV-miR-320c was again found to be associated with CRC recurrence (Fig. 4L). Together, our clinically oriented integrative analysis provided strong support to the versatile use of EV-miR-320c in discriminating particular disease status and outcome.

**Table 5:** Overall survival-associated EV-miRs (stages I-IV)

OS associated EV-miRs	p.value
EV-miR-320c	p = 0.0016
EV-miR-30b-3p	p = 0.0096
EV-miR-4665-5p	p = 0.01
EV-miR-193b-5p	p = 0.011
EV-miR-330-3p	p = 0.012
EV-miR-106a-5p	p = 0.012
EV-let-7f-1-3p	p = 0.013
EV-miR-200c-3p	p = 0.015
EV-miR-200b-3p	p = 0.026
EV-miR-320d	p = 0.027
EV-miR-199b-5p	p = 0.029
EV-miR-3679-5p	p = 0.033
EV-miR-942-5p	p = 0.034
EV-miR-23b-5p	p = 0.034
EV-miR-1301-3p	p = 0.037
EV-miR-23a-3p	p = 0.037
EV-miR-4510	p = 0.038
EV-miR-548j-5p	p = 0.041
EV-miR-338-5p	p = 0.043
EV-miR-199b-3p	p = 0.046

**Table 6:** DFS-associated EV-miRs (stages I-III)

PFS associated EV-miRs	p.value
EV-miR-942-5p	p = 0.007
EV-miR-582-3p	p = 0.0073
EV-miR-221-5p	p = 0.0073
EV-miR-1304-3p	p = 0.01
EV-miR-203a-3p	p = 0.011
EV-let-7f-1-3p	p = 0.015
EV-miR-320c	p = 0.023
EV-miR-181d-5p	p = 0.026
EV-miR-660-5p	p = 0.026
EV-miR-370-3p	p = 0.029
EV-miR-210-3p	p = 0.029
EV-miR-181a-5p	p = 0.035
EV-miR-18a-3p	p = 0.037
EV-miR-219a-1-3p	p = 0.041

### Characterization of miR-320c in the CRC primary tumors and cell lines indicates functional and clinical distinction of the circulating EV-miR-320c

**Fig. 5:**
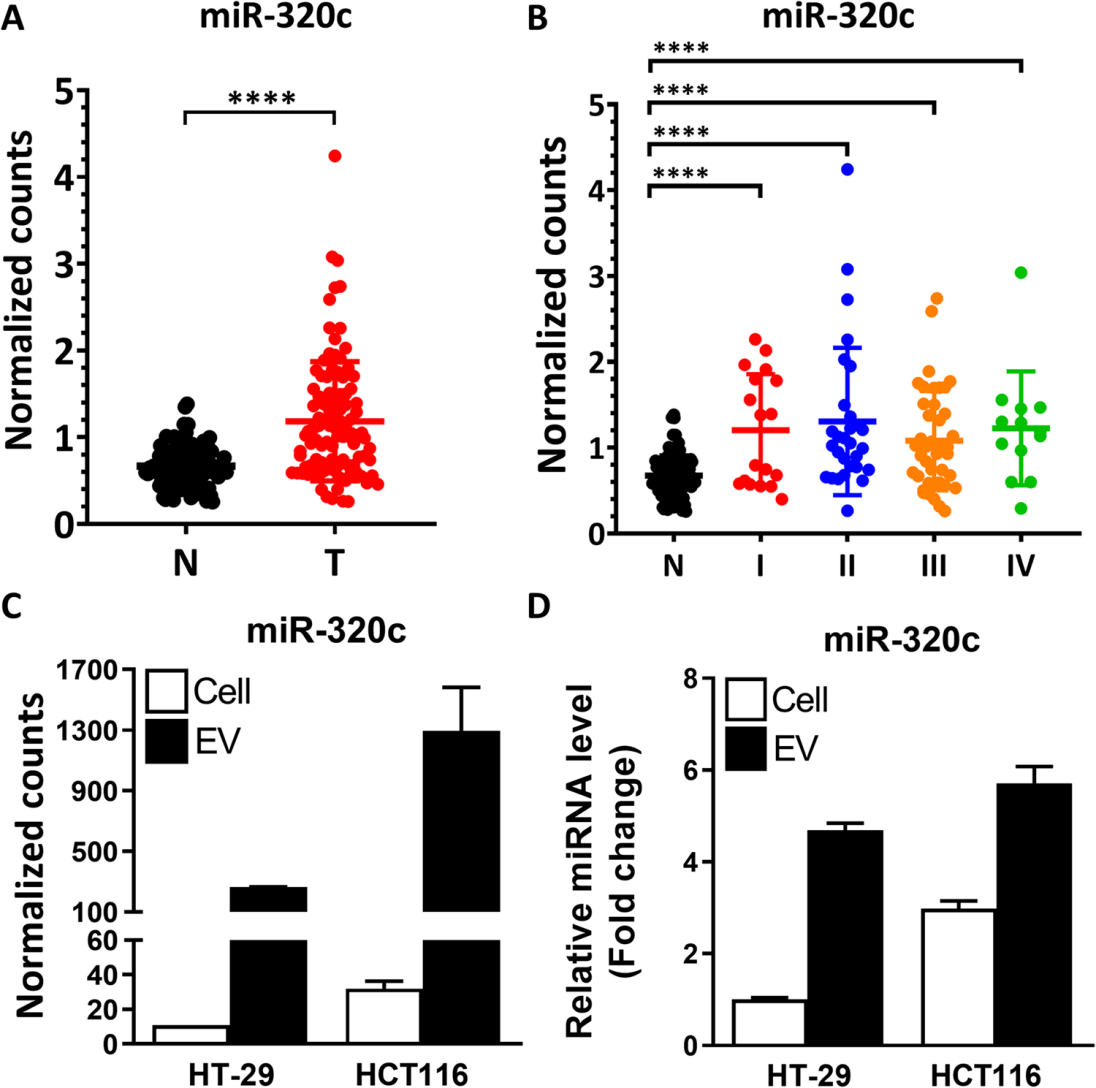
The expression levels of miR-320c in the CRC primary tumor vs. adjacent normal tissues and in cultured CRC cells. (**A**) Distribution of miR-320c expression levels, based on small RNA-seq data, in the 102 pairs of CRC tumor and adjacent normal tissues. (**B**) Paired specimens were further grouped into four clinical stages, and miR-320c expression patterns across the different stages of CRC are depicted. (**C & D**) The expression patterns of miR-320c in the cell extracts (Cell) and EVs isolated from medium, as determined by small RNA-seq (**C**) and RT-qPCR (**D**) analyses. Two CRC cell lines, HT-29 and HCT116, were monitored

Recently, we have completed sequencing and data analysis of transcriptomes and miRNA profiles in 102 pairs of Taiwan CRC primary tumor and adjacent normal tissue specimens (Table 7) [10]. In these tissue datasets, we detected 1,671 types of miRNAs, 862 of which showed detectable expression in more than 50% of the tissue samples. On average, 958 miRNAs were identified in each tumor tissue sample, whereas the adjacent normal controls expressed 921 types of miRNAs (p = 0.0026, Fig. S3B), again indicating a tumor-associated alteration in miRome landscape. Given the presence of circulating EV-miR-320c as revealed above, we examined the expression patterns of miR-320c in the transcriptomes of CRC primary tumors. Our analysis showed that miR-320c was significantly upregulated in the tumor tissues in comparison to adjacent regions (Fig. 5A), but did not show a significant difference between samples across different stages (Fig. 5B). Next, we also carried out miRome-wide analysis for the association of the individual miRNAs with patient survival and recurrence outcome. In the CRC tissue miRome datasets, there were 102 patients with overall survival (stage I-IV) and 89 with recurrence data (stage I-III). We then uncovered 56 and 57 miRs with significant connection to patient overall survival and recurrence-free survival, respectively (Kaplan and Meier analysis, p < 0.05, Tables S5 and S6). However, miR-320c was not identified as one of the tissue candidate markers for patient survival and disease recurrence. These results indicated that the miR-320c detected in the primary tumor tissues of CRC might not be functionally and pathologically equivalent to the EV-miR-320c present in the circulating small EVs.

**Table 7:** Characteristics of the CRC tissue samples in the study

Clinical stage (TNM)	CRC (n = 102)			
	I	II	III	IV
Patient numbers	18	29	42	13
Age in years, mean (SD)	64.11(8.61)	62.07(11.39)	60.57(10.83)	60.54(8.82)
Range	51–79	39–79	39–85	46–73
Gender, n (%)				
Female	3(16.7)	12(41.4)	23(54.8)	6(46.2)
Male	15(83.3)	17(58.6)	19(45.2)	7(53.8)

To further demonstrate that the identified mCRC-EV-miRs were functionally distinct entities, we next sought to confirm that these miRs were indeed derived from the extracellular vesicles secreted by CRC cells. To test this hypothesis, we carried out small RNA sequencing and RT-qPCR analyses on RNA prepared from both the cells and media/EVs of two CRC lines, HT-29 and HCT116. Our results showed that miR-320c, as well as the majority of the 15 mCRC-EV-miRs, exhibited abundant expression in the EV fraction of these two cell lines (Fig. 5C & D, and Supplementary Fig. S4). These observations implied that our candidate miRNAs were originated from the extracellular vesicles secreted from the CRC cells.

In silico analyzed the function of the 15 mCRC-EV-miRs indicates their involvement in MET and niche programming.

**Fig. 6:**
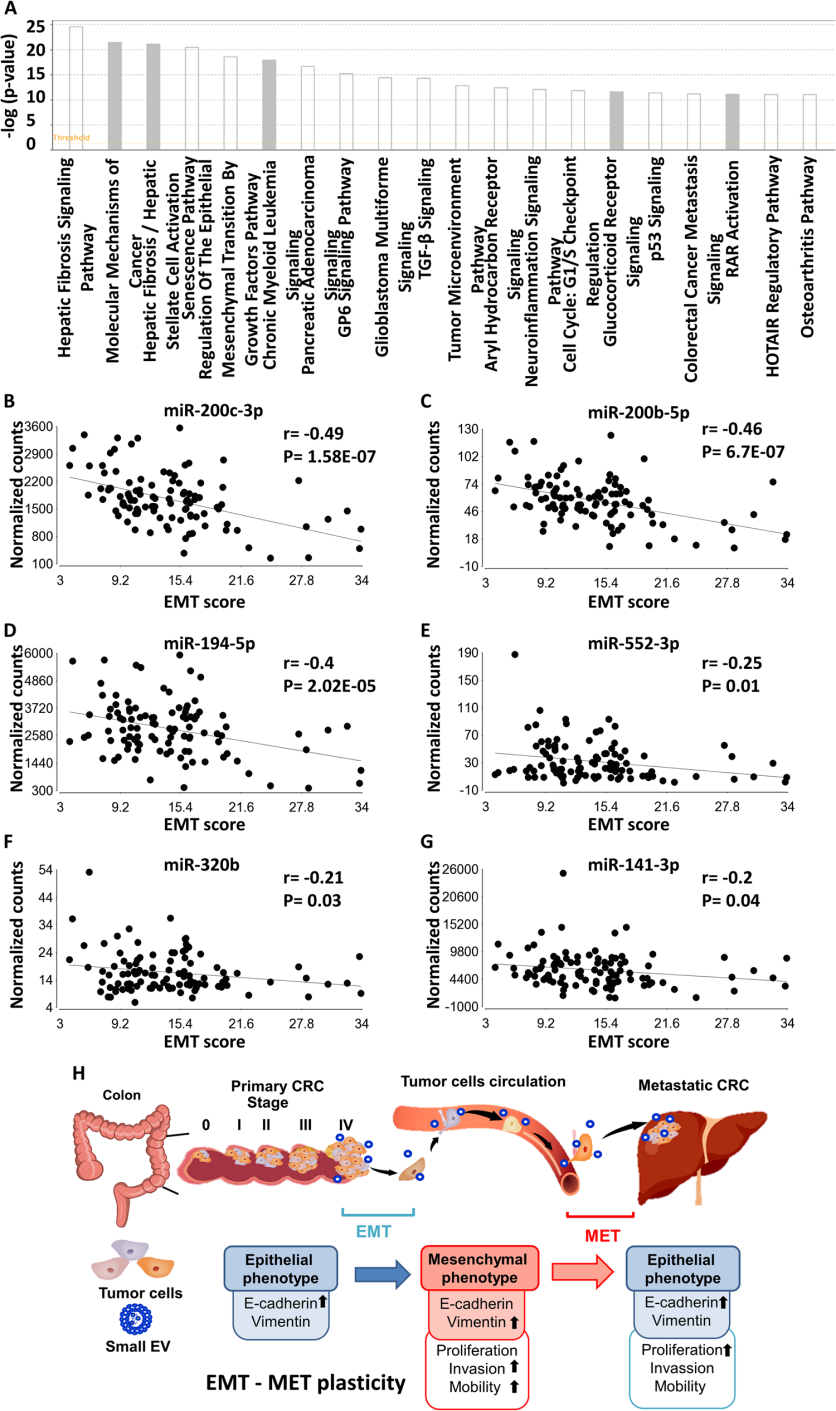
The impact of 15 mCRC-EV-miRs on MET in the metastasized cells. (**A**) 165 mRNA gene targets potentially under regulation by the 15 mCRC-EV-miRs were mapped based on prior experimental evidence (microRNA Target Filter analysis module of IPA), functional enrichment of which were further uncovered by the Core Analysis of IPA (top 20 canonical pathways are shown). The colors of the bars correspond to the possibility of whether the activity of the enriched pathway could be predicted (white, yes; gray, no). (**B-G**) Cohort-wide correlation of miRNA expression with EMT scores. Expression levels of the indicated miRs, respectively miR-200c-3p (**B**), miR-200b-5p (**C**), miR-194-5p (**D**), miR-552-3p (**E**), miR-320b (**F**), miR-141-3p (**G**), in each patient were analyzed for the extent of correlation with the respective EMT score, as shown in the dot plots. Correlation coefficient and statistical significance of each comparison are also denoted. (**H**) Schematic depiction of the potential involvement of progression-dependent circulating EV-miRs in reprogramming the metastasized cells into a metastasis-favorable MET state

To provide functional evidence for the panel of 15 mCRC-EV-miRs identified with dual detection and monitoring roles (Table 3), we set out to search for their possible cellular gene targets by using the IPA microRNA Target Filter analysis. We found that these mCRC-EV-miRs could target 165 mRNA genes on the basis of previously annotated experimental evidence (Table S7). Further IPA canonical pathway analysis of these candidate mRNAs, as shown in Fig. 6A (Top 20) and Table S8, revealed an enrichment of genes with functional implications in several key cellular processes: (1) EMT pathways related to TGF-β, Wnt/β-catenin, and HIF-1α signaling; (2) tumor microenvironment, macrophage, and natural killer cell signaling; (3) inhibition of angiogenesis by TSP1; and (4) PTEN and apoptosis pathways.

Notably, among the 15 mCRC-EV-miRs, 7 EV-miRs (EV-miR-320c, EV-miR-29a-3p, EV-miR-24-3p, EV-miR-141-3p, EV-miR-193a-5p, EV-miR-200c-3p, and EV-miR-222-3p) were functionally implicated in the EMT pathways based on the target spectrum (Table S9). However, since the levels of these EV-miRs were found by our profiling to be upregulated in the metastatic CRC patients, and miRNAs typically negatively impact target genes, we postulated that, rather than acting to promote the EMT in the CRC cells, these metastatic EV-miRs likely might mediate the MET in the metastasized cells. EMT is a reversible process through which cells lose their epithelial characteristics, for instance, cell polarity and cell–cell contact, and subsequently gain mesenchymal properties, such as increased motility. Given the molecular basis of the state transition during EMT, it can be quantitatively characterized by the gene expression-based EMT score, which is positively correlated with the mesenchymal state of the cells [13]. To further delineate whether our identified mCRC-EV-miRs are functionally connected to EMT progression in CRC, we then turned to our published mRNA and miRNA sequencing datasets on 102 CRC tissues [10] and searched for coordinated patterns between the expression of candidate EV-miRs and the EMT scores of tumor tissues. Among the 15-miR panel, Pearson’s correlation uncovered six miRs with a significantly inverse relationship to the EMT scores within our CRC cohort (miR-200-3p, miR-200b-5p, miR-194-5p, miR-552-3p, miR-320b, miR-141-3p) (Fig. 6B to G). These observations imply that these miRNAs, upon EV uptake by the metastasized tumor cells, might mediate a cellular state amenable to the MET and consequently the stabilized development at the secondary niche. Their strong inverse correlations with the EMT scores then suggest that these miRNAs might control processes in parallel with and/or downstream of the acquisition of the pro-metastatic mesenchymal state. Taken together, these in silico functional annotation and analyses of the EV-miRs-centric networks provided strong evidence that mCRC-EV-miRs might participate in programming the MET for the metastasized CRC cells to settle at the secondary site and also in creating a tumor-favoring metastatic niche (Fig. 6H).

## Discussion

In this study, we established and optimized a rapid, sensitive, and robust liquid biopsy sampling method. By cross-comparison of EV-miRomes (n = 290) from multistage and longitudinal cohorts, our clinically oriented analysis further delineated a 15-EV-miR signature with dual detection and monitoring functions for mCRC. Furthermore, the target spectrum of this 15-EV-miR signature of mCRC suggested an involvement of small EVs in the tumor microenvironment and programming of the MET for distant localization of the metastasized cells and in creating a tumor-favoring metastatic niche (Fig. 6H). EV-miRs are reportedly involved in altering tumor state, progression, and metastasis [14]. In this capacity, several EV-miRs from CRC cells (miR-25-3p, miR-130b-3p, and miR-425-5p) have been found to induce the tumor-supporting M2 polarization of macrophages (tumor-supporting macrophages) via regulating the PI3K/Akt-PTEN signaling pathway [15]. Other examples of metastasis-related EV-miRs include tumor-derived EV-miR-1247-3p, which is known to induce cancer-associated fibroblast activation to foster lung metastasis of liver cancer [16]; EV-miR-25-3p, which promotes extravasation and vascular permeability in the liver and lungs in a mouse model of CRC metastasis, through targeting of the transcription factors Krüppel-like factors 2 and 4 [17]; and EV-miR-214, which upon delivery to mouse peripheral CD4 + T cells downregulates PTEN and promotes Treg expansion [18]. Cancer cell EV-miRs can also block the adaptive immune response by affecting natural killer cells or by decreasing dendritic cell maturation [19]. While strong evidence supports the critical function of small EVs in tumor cell dissemination via EMT induction, there are also several conceptual gaps regarding their roles in metastatic outgrowth, such as metastatic reactivation or MET transition for the settlement of metastasized cells at a secondary site [20].

EMT is a reversible process through which cells lose their epithelial characteristics, and subsequently gain mesenchymal properties, such as increased motility. This dynamic cellular transition is hypothesized to be co-opted by carcinoma to confer an invasive or metastatic phenotype on tumor cells and is reportedly driven by multiple signaling pathways in CRC: (1) The TGF-β signaling, linked to tumor progression, stimulates the expression of EMT markers such as SNAIL, vimentin, and fibronectin. (2) Overactivation of the WNT/β-catenin pathway promotes EMT-associated dedifferentiation at the invasive front of CRC tumors. (3) The hypoxia-responsive HIF-1α induces EMT and cell invasion by mediating activation of ZEB1. Interestingly, the possibility that contents of extracellular vesicles could facilitate the reversion of tumor state in favor of metastasis has been illustrated by several recent reports [20, 21]. Melanoma small EVs have been demonstrated to educate bone marrow progenitor cells toward a pro-metastatic phenotype through mesenchymal-to-epithelial (MET) [21], while small EVs-mediated citrullination of extracellular matrix is implicated in the mesenchymal-to-epithelial transition and liver metastasis [20]. Multiple lines of evidence have also pinpointed the exosomal miR-200 family as a biomarker for tumor prognosis and metastasis prediction and further demonstrated that exosomal miR-200c and miR-141 are under control by the MET signaling [22–24]. Accordingly, the abundance of cellular miR-200 in liver metastasis tissue is much higher than in the primary tumor tissue, and this miRNA is known to target ZEB1 and enhance subtype transition from M to E in the metastasized cells [25]. Interestingly, miR-200 family is also a candidate in our 15 mCRC-EV-miR signatures (Table 3: EV-miR200b-5p, EV-miR200c-3p, and EV-miR141-3p). Moreover, in terms of our candidate EV-miR-320c, the miR-320 family also exhibits high expression in the serum of metastatic rectal cancer [26] and was found to negatively impact multiple signaling pathways upstream of the EMT process [27]. Our clinically oriented delineation of the 15-mCRC-EV-miRs, further support the function of small EVs in programming the mesenchymal–epithelial transition (MET) for distant localization of the metastasized cells.

Altered expression profiles of circulating miR-320c in colorectal cancer has been documented by other groups, despite limited information on its diagnostic utility. Wang et al. reported the upregulation of miR-320c in plasma exosomes of the Chinese patients with early stage colon cancer (stage I/II); however, the diagnostic power of this marker in distinguishing disease state was low (AUC = 0.5982) [3]. Another Norway group also demonstrated that miR-320c was upregulated in the serum from metastatic rectal cancer patients, despite no clinical marker evaluation [26]. Based on the data archived at the publicly available EVmiRNA database (http://bioinfo.life.hust.edu.cn/EVmiRNA) [28], miR-320c was indeed highly expressed in the extracellular vesicles in the colon carcinoma in comparison with the other malignancies (Supplementary Fig. S5), suggesting that its elevation in liquid biopsy might indeed be a clinically relevant phenomenon. In addition to the expression alteration, our present findings conclusively demonstrated that EV-miR-320c could serve as a powerful indicator of multiple clinical attributes, such as mCRC detection and real-time monitoring of treatment response. To our knowledge, our study is the first report on the potential translational application of EV-miR-320c for colorectal cancer.

Teng et al. have recently demonstrated in the mouse tumor model that the sorting of oncomiRs (oncogenic microRNA) into exosomes is suppressed, while the sorting of TS-miRs (tumor suppressor microRNA) is increased [29]. Furthermore, they found that TS-miRs including miR-193a are significantly higher in patients with liver metastasis than in non-metastatic CRC. Consistent with our findings, our 15 mCRC-EV-miR signatures are also enriched in TS-miRs, including the miR-320 family, miR-200 family, and miR-193a. Many studies have confirmed that miR-320 is downregulated during tumorigenesis and could serve as a crucial suppressor of tumor proliferation and metastasis [30–33]. Several lines of findings have also shown that miR-320 family members were negatively regulated in the process of tumor cell migration and invasion [27]. Interestingly, Hong et al. found that miR-320 antagonizes EMT, a function mediated by directly targeting forkhead box M1 (FOXM1) that leads to upregulated E-cadherin and suppressed N-cadherin and vimentin [30]. Moreover, the downregulation of miR-320 has been implicated in tumor resistance to therapeutic drugs such as oxaliplatin, epirubicin, gemcitabine, tamoxifen, and doxorubicin [34–40]. The recovery of miR-320 expression was demonstrated by various reports to effectively alleviate tumor resistance to certain drugs [34, 35, 39]. However, the mode of action and mechanism underlying miR-320-mediated drug resistance is not fully resolved. In addition, recent studies have also supported that, rather than acting as a tumor repressor, miR-320 could also be positively correlated with tumorigenesis [41–43], suggesting that its pro-tumoral or anti-tumoral functions may be context-dependent. Despite the documented roles of miR-320c in the cells, no studies have yet pointed out the impact of circulating EV-miR-320c on the process of tumor formation. According to our findings, EV-miR-320c is presumed to participate in programming the MET for the CRC cells undergoing distant metastasis and also in creating a tumor-favoring metastatic niche (Fig. 6H). Functional identification of circulating EV-miR-320c downstream gene regulatory network in the recipient cells in the colorectal cancer metastatic progression and microenvironment dynamics constitutes a noteworthy direction of further research efforts.

## Supplementary Information

Below is the link to the electronic supplementary material.

Supplementary file1 (XLSX 51 KB)

Supplementary file2 (PDF 345 KB)

## Data Availability

The small RNA sequencing data of the 290 EV-miRomes generated in this study were deposited at NCBI Gene Expression Omnibus (GEO) database under the accession code GSE188627. The small RNA sequencing and RNA sequencing data of the CRC tissues were reported previously and are available publicly in NCBI Sequence Read Archive (SRA) database with the project accession number PRJNA387172.

## Abbreviations

EV: Extracellular vesicle
mCRC: Metastatic colorectal cancer
CEA: Carcinoembryonic antigen
MET: Mesenchymal–epithelial transition
EMT: Epithelial-mesenchymal transition
ROC: Receiver operating characteristic
AUC: Area under the receiver operating characteristic curve
