# Supplementary material for: EV-miRome-wide profiling uncovers miR-320c for detecting metastatic colorectal cancer and monitoring the therapeutic response
Source: Cell Oncol (Dordr). 2022 Jul 18;45(4):621–38. doi: 10.1007/s13402-022-00688-3 (PMC9424175; doi:10.1007/s13402-022-00688-3)
Supplement: Supplementary file 2 — Supplementary file2 (PDF 345 KB) [file 13402_2022_688_MOESM2_ESM.pdf]

Supplementary Figure S1

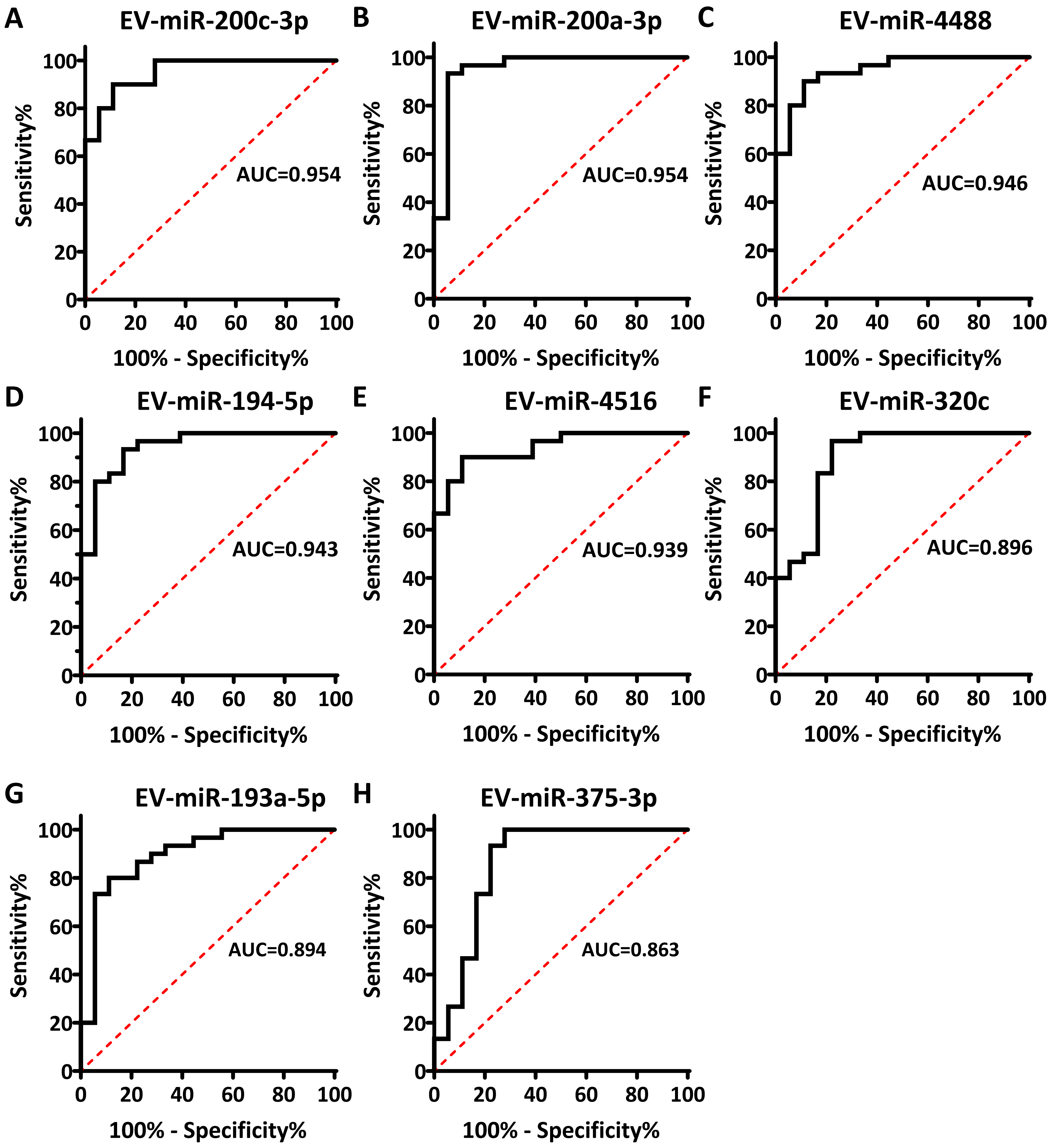

Supplementary Figure S1. Strong early diagnostic value of candidate EV-miRs.
(**A** to **H**) In evaluating the early diagnostic value of these candidate EV-miRs, the ROC curve analysis revealed five EV-miRs with AUC values of more than 0.9 (**A** to **E**, respectively, EV-miR-200c-3p, EV-200a-3p, EV-miR-4488, EV-miR-194-5p, and EV-miR-4516) and three additional EV-miRs exhibiting AUC of >0.85 (**F** to **H**, respectively, EV-miR-320c, EV-miR-193a-5p, and EV-miR-375-3p) (also see Supplementary Table S2).

Supplementary Figure S2

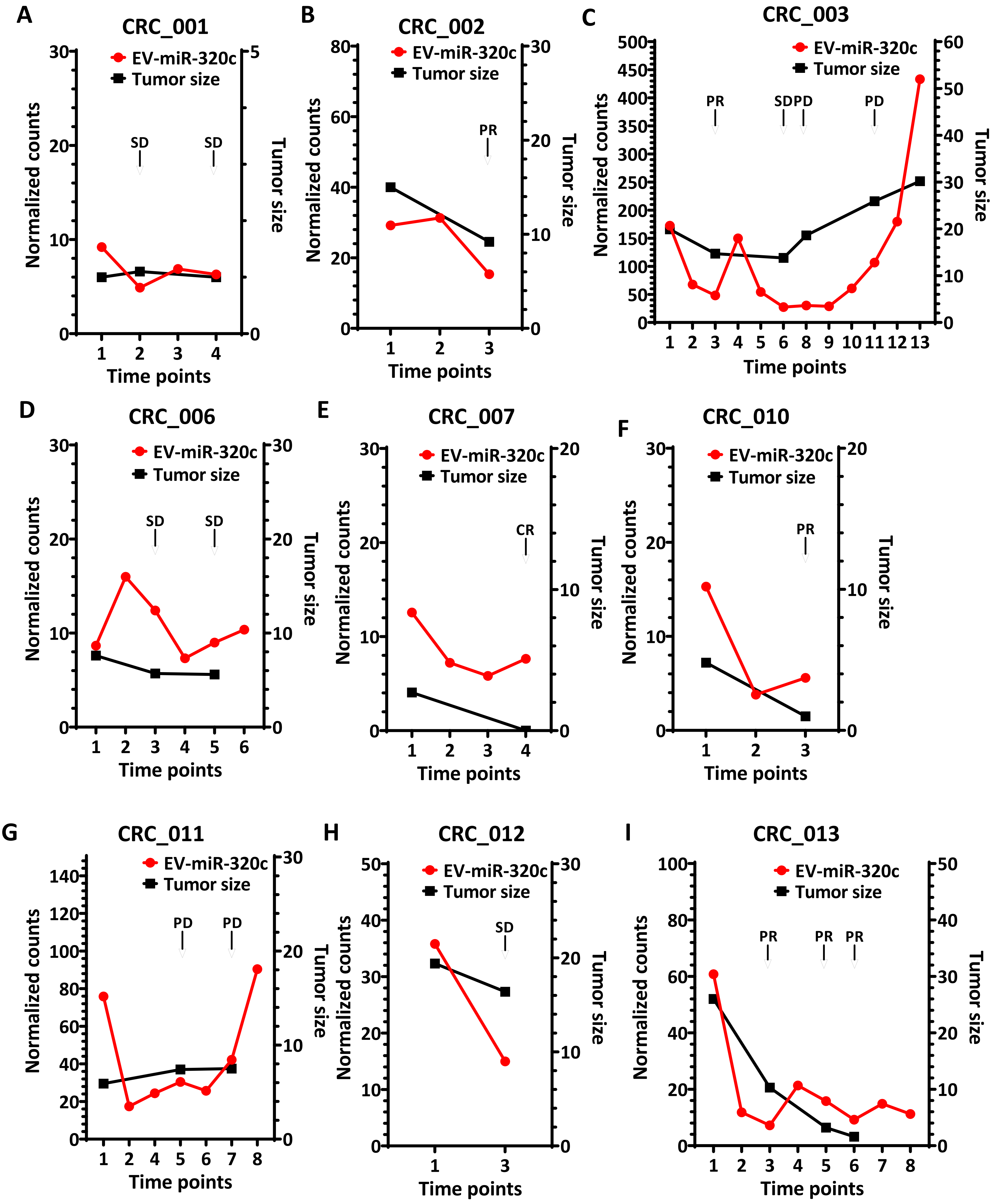

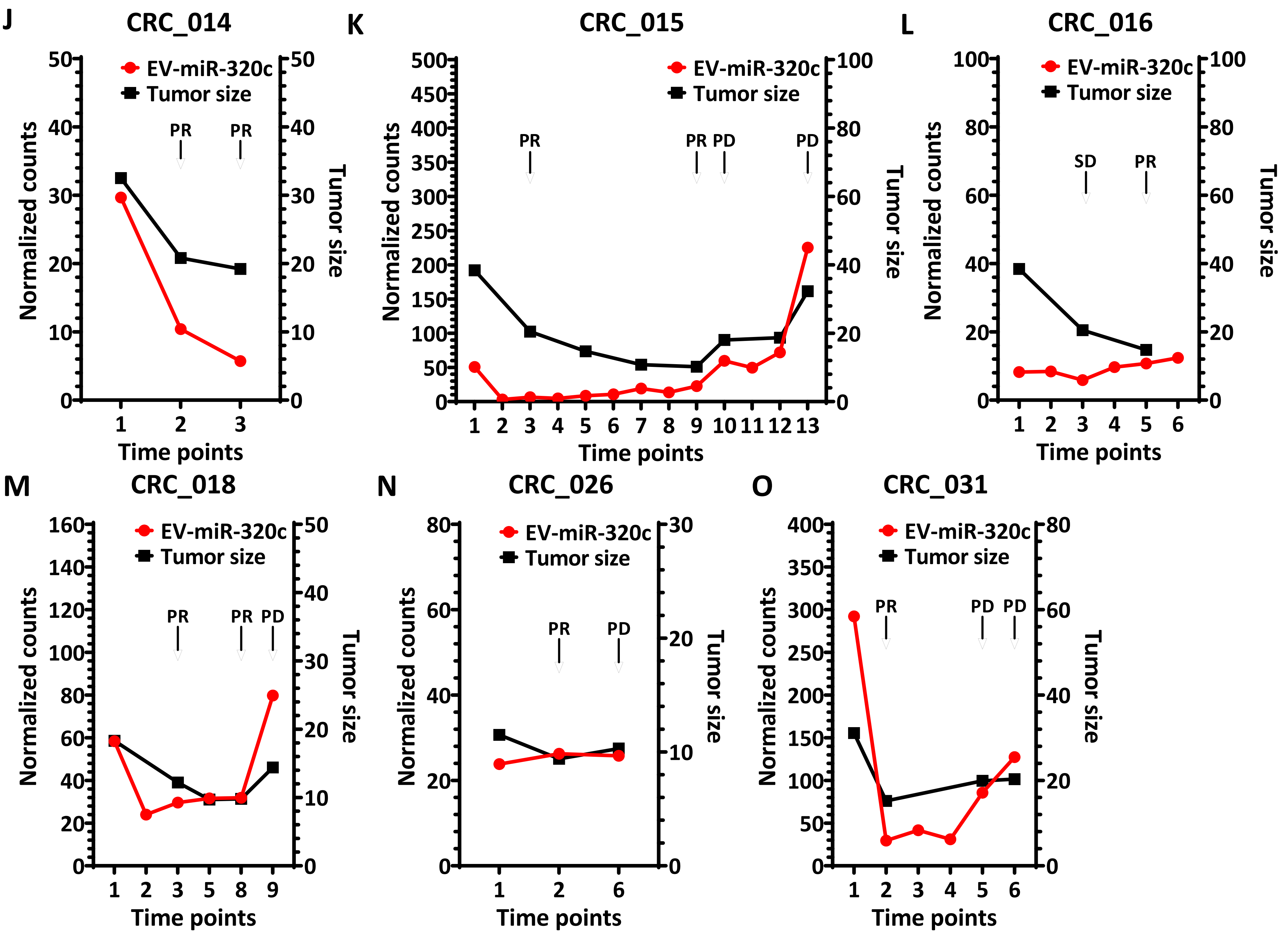

Supplementary Figure S2. Correlation between EV-miR-320c expression and patient tumor size.
(**A** to **O**) The coordinated changes in EV-miR-320c expression (left axis; red curve) with the tumor sizes (right curve; black curve) in the selected 15 mCRC patients are plotted and shown. Refer to Figure 3E.

Supplementary Figure S3

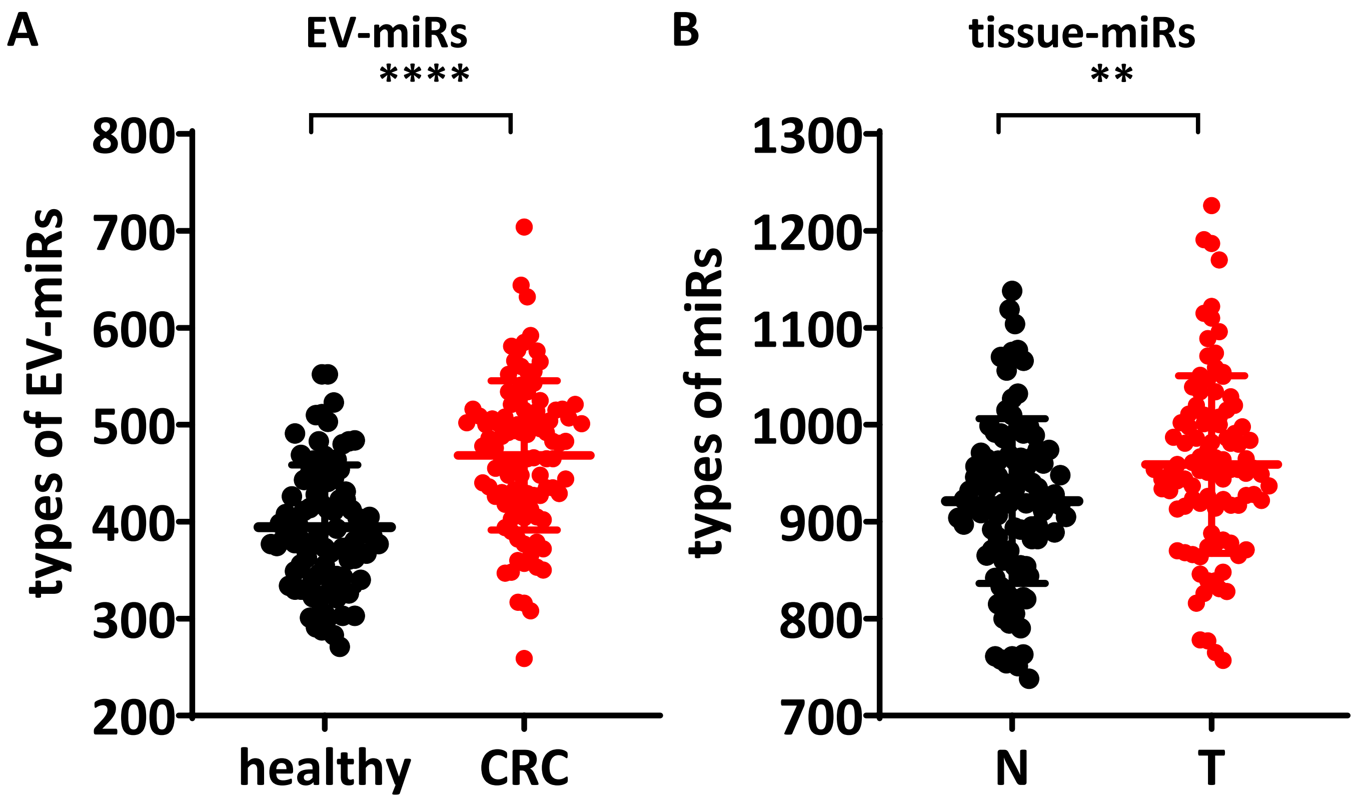

Supplementary Figure S3. Comparative distributions of distinct miRNA types expressed in small EVs vs. tumor tissues. The number of distinct miRNA genes identified in each of the plasma samples of healthy subjects vs. CRC patients (**A**) and of the specimens of adjacent normal vs. tumor tissues (**B**) are shown.

Supplementary Figure S4

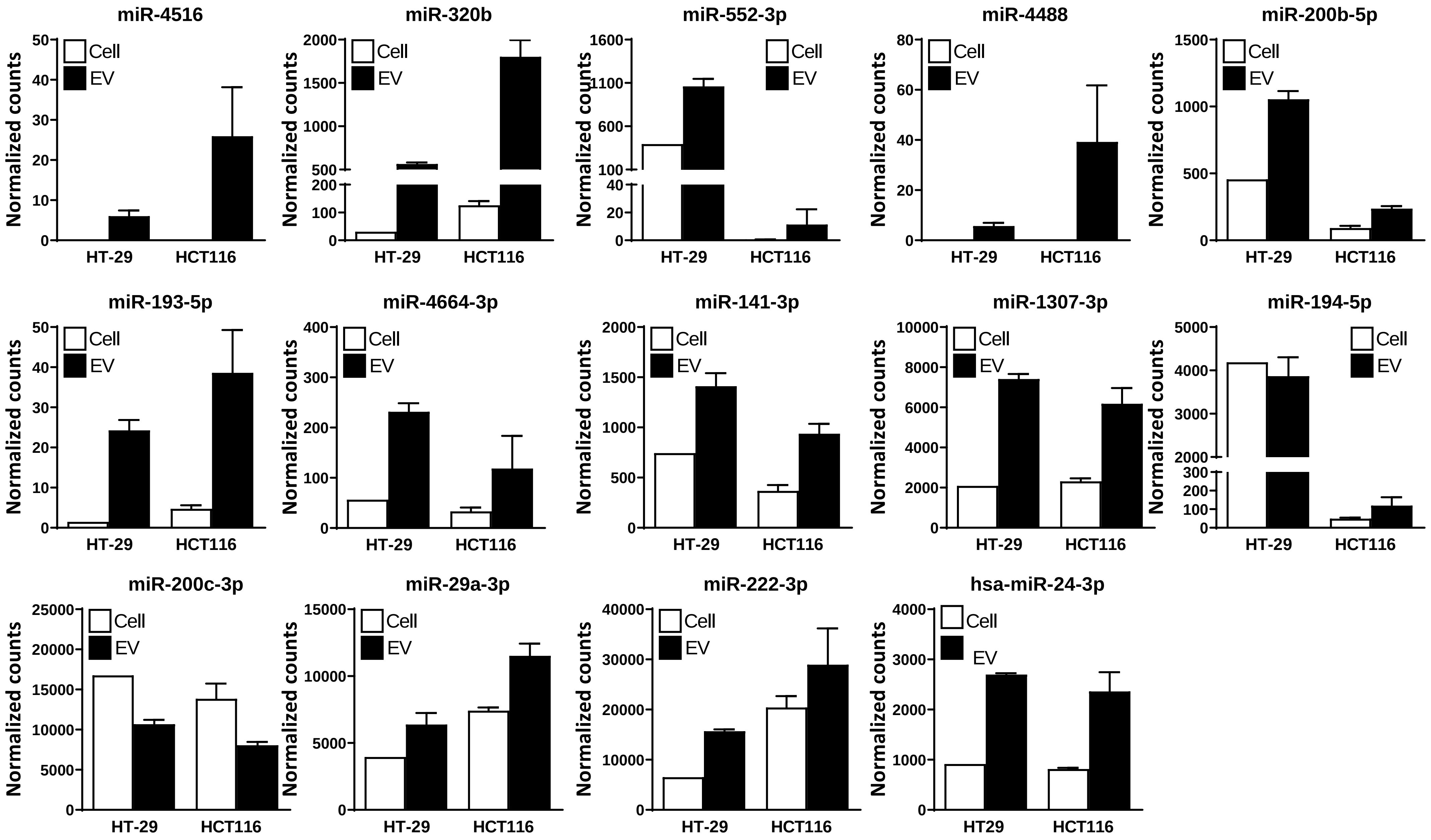

Supplementary Figure S4. The expression levels of candidate miRNAs in cultured CRC cells. The expression patterns of the indicated miRNAs (as shown in Table 3) in the cell extracts (Cell) and EVs isolated from medium, as determined by small RNA-seq analyses. Two CRC cell lines, HT-29 and HCT116, were monitored. These results correspond to Figure 5, C & D.

Supplementary Figure S5

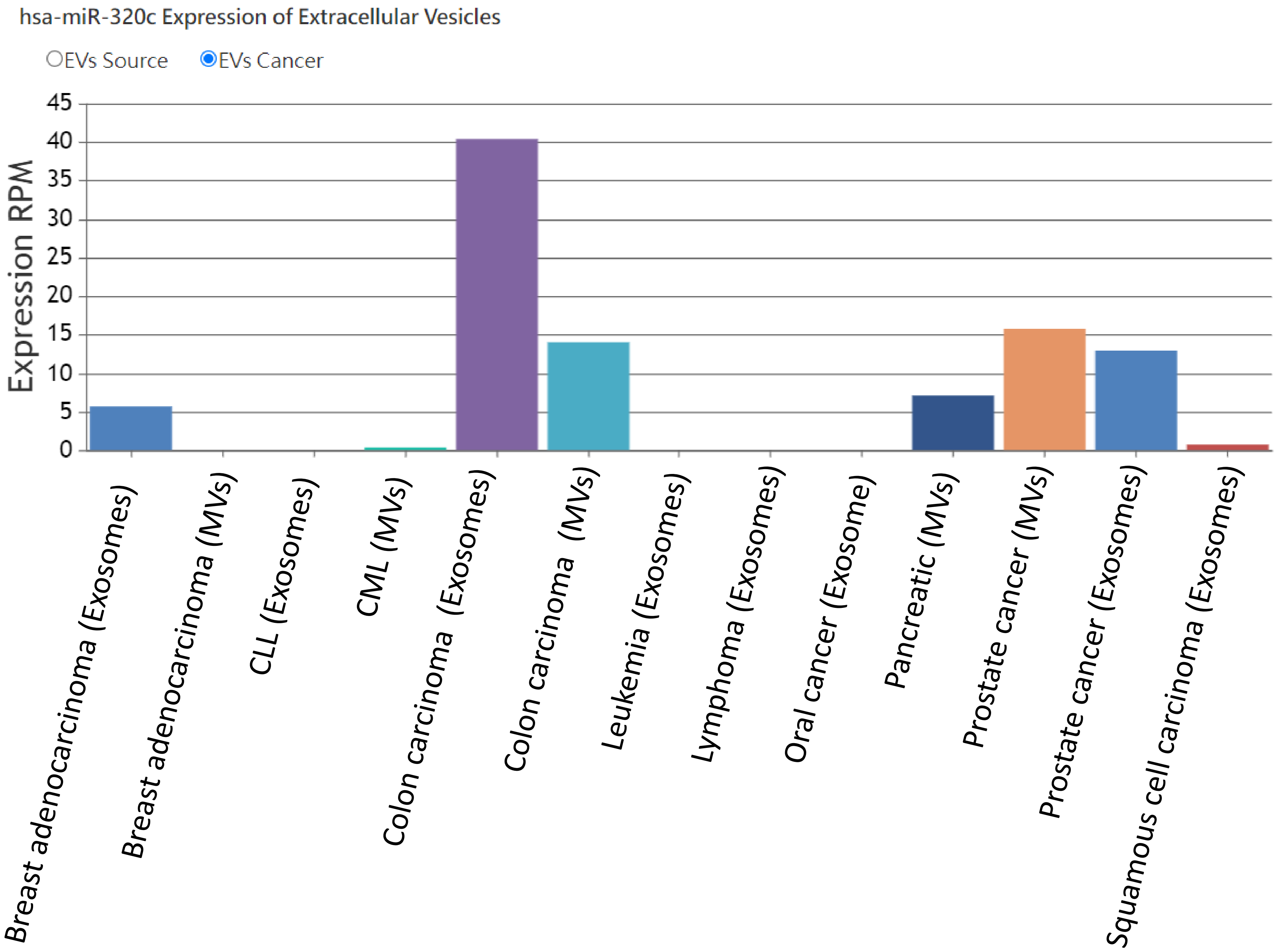

Supplementary Figure S5. Expression spectrum of circulating miR-320c in the liquid biopsies of different malignancies. The expression levels of miR-320c, based on normalized sequencing reads, in the extracellular vesicles isolated from the indicated cancer types are shown. The bar graph depicts data archived at the publicly available EVmiRNA database (http://bioinfo.life.hust.edu.cn/EVmiRNA) [28], and is adapted from the website.
